# Role of αBI5 and αBT162 residues in subunit interaction during oligomerization of αB-crystallin

**Published:** 2008-10-16

**Authors:** Raju Murugesan, Puttur Santhoshkumar, K. Krishna Sharma

**Affiliations:** Departments of Ophthalmology and Biochemistry, University of Missouri, Columbia, MO

## Abstract

**Purpose:**

To determine whether the residues in the NH_2_- and COOH-terminal extensions interact with one another during oligomerization of αB-crystallin.

**Methods:**

Site-directed mutagenesis was used to mutate αBI5 and αBT162 residues to Cys. The recombinant I5C and T162C proteins were expressed in *Escherichia coli* cells and purified using chromatographic techniques. These proteins were analyzed by SDS–PAGE and mass spectrometry and characterized by multi-angle light scattering and circular dichroism (CD) spectroscopy methods. Fluorescence resonance energy transfer (FRET) assay was used to determine the interaction between the subunits.

**Results:**

Dimer formation was observed in both αBI5C and αBT162C in storage at 4 °C. During air oxidation at room temperature, αBT162C formed dimers to a greater extent than αBI5C. The average molar masses, secondary structures, and chaperone-like activities of the reduced forms of I5C and T162C were comparable to that of wild type αB-crystallin. The oligomeric assembly of reduced forms of I5C and T162C appeared homogenous under JEOL 1200EX Electron microscope whereas the oxidized proteins appeared as irregular aggregates. FRET assay demonstrated interactions between αBI5C–αBI5C and αBT162C–αBT162C. However, there was no evidence of an interaction between αBI5C and αBT162C residues during oligomerization.

**Conclusions:**

This study suggests that residues from the NH_2_- and COOH-terminal regions in αB-crystallin interact with residues from the corresponding regions of another subunit, but there exists no interaction between the residues at the COOH-terminal extension region and the residues at the NH_2_-terminal region.

## Introduction

Human αB-crystallin, also known as HspB5 [1], is a small heat-shock protein (sHSP) that contains 175 amino acids and has a molecular mass of 20 kDa [2]. αB-Crystallin has chaperone-like properties [3], is expressed in various tissues, and responds to thermal, environmental, and chemical stressors. Upregulation of αB-crystallin has been reported in Alzheimer and Parkinson diseases [4], and mutant αB-crystallin has been associated with cataract formation and desmin-related myopathy [5,6]. αΒ-Crystallin is an intensively studied lens protein yet its three-dimensional structure remains unknown. Its polydispersity is the main obstacle to the acquisition of X-ray crystallographic data. Further, the crystallin oligomer is too bulky for structure determination by nuclear magnetic resonance spectroscopy [7]. To circumvent these limitations, several solution-based interaction studies and low-resolution electron microscopy (EM)–based structural studies have been conducted but have provided limited clues to the structural organization of the αB-crystallin oligomer and its hetero-oligomer with αA-crystallin [8,9].

Sequence comparison with other sHSPs shows that αB-crystallin has an evolutionarily conserved sequence with a crystallin domain of several β strands in an immunoglobulin-like fold flanked by NH_2_- and COOH-terminal extensions [10,11]. The X-ray crystal structures of related proteins, Mj sHSP16.5 (*Methanococcus jannaschii* sHSP16.5) and wheat sHSP16.9, suggest that there exist common structural features that can be attributed to the α-crystallin domain region in these proteins [12-14]. In αB-crystallin, residues 19–71, 75–82, 131–138, 141–148, and 155–164 have been implicated in oligomerization and subunit interactions [15-19].

The COOH-terminal of sHSPs is largely unstructured, contains many charged residues, and is believed to play a role in solubilization [7,11,14]. The results obtained from the mammalian two-hybrid system has shown that the COOH-terminal region was important for αB-crystallin oligomerization [20], and the results were confirmed by other studies based on peptide-protein interaction [21] and change in oligomer size following truncation [22] or mutations [23]. The COOH-terminal 157–164 region of αB-crystallin has been shown to be a chaperone site [17], and deletion of this region resulted in poor solubility and larger aggregate formation, which suggests that this region is critical for the function of the protein [19]. Site directed mutagenesis studies of conserved IXI/V motif in the COOH-terminal extension of αB-crystallin have shown that this region comes in the proximity of the β8-strand in the α-crystallin domain and also interacts with the same motif from another subunit [24]. However, the involvement of residues COOH-terminal to the IXI/V motif in oligomerization is not fully understood. Lys150 of αB-crystallin was found to cross-link with Lys166 of either the same or another molecule of αB-crystallin when 3.3′-dithiobis(sulfosuccinimidyl propionate) was used as a cross-linker [25]. It is not clear to what extent the cross-linker length was a determining factor in this presumed interaction. Protein cross-linking studies [25,26] have not shown NH_2_-terminus interaction between αB-crystallin subunits. A pin array study showed a weak interaction between αB-crystallin and immobilized residues 11–18 [27], but this has not been confirmed by other studies. In sHSP 16.9, on the basis of crystal structure, it has been concluded that only the NH_2_-terminal region of six subunits in an oligomer has some structure whereas the other six subunits NH_2_-terminal regions are presumed disordered. Further, it has also been shown that the NH_2_-terminal residues 7–10 in HSP 16.9 interact with residues 108–110 in the subunit and act as a “patch” [13]. Residues 7–10 of HSP 16.9 are analogous to residues 21- 24 in αB-crystallin [13] since the αB-crystallin NH_2_-terminal region has an additional 14 residues compared to HSP 16.9. Previous mutagenesis or two-hybrid system studies have not addressed the role of NH_2_-terminal residues 1–19 in homo-oligomerization of αB-crystallin. The αB-crystallin oligomer is a dynamic molecule that constantly dissociates and reassociates [28-30]. Since the COOH-terminal truncation of αB-crystallin has been observed during aging and diabetic lenses [31], it has been proposed that this modification might be affecting the oligomer nature and function of α-crystallin in vivo.

Oxidation of single cysteine proteins leads to dimerization when the reactive cysteines are in close proximity. Therefore, to investigate the role of the NH_2_-terminal region in αB-crystallin oligomerization in the present study, we converted the I5 residue to Cys and characterized the recombinant protein. The I5 residue is outside the αB-crystallin sequence that is homologous to sHSP 16.5 and sHSP 16.9, of which the crystal structure is known [12,13]. We also prepared a T162C mutant of αB-crystallin to determine the role of COOH-terminal extension outside the conserved IXI/V motif in oligomerization. The extent of interactions between the NH_2_-terminal and COOH-terminal regions of mutant αB-crystallin was investigated using the fluorescence resonance energy transfer (FRET) assay. Our study showed that the I5 residue in the NH_2_-terminal region is involved in subunit interaction to a lesser extent than the T162C residues in the COOH-terminal extension. Additionally, there was no evidence of interactions between I5C and T162C residues in the subunits.

## Methods

### Expression and purification of recombinant proteins

Human αB-crystallin cDNA was cloned in pET-23d(+) vector (Novagen-EMD Biosciences, Madison, WI) and used as the template to introduce cysteine mutations at αBI5 or αBT162 by site-directed mutagenesis (Quik-Change site-directed mutagenesis kit, Stratagene, La Jolla, CA). Mutation was confirmed by automated DNA sequencing. Wild-type and mutant proteins were expressed in *Escherichia coli* BL21 (DE3) pLysS cells (Invitrogen, Carlsbad, CA) and purified according to the method described previously [32]. Purity of the recombinant proteins was evaluated by SDS–PAGE, and molecular mass was confirmed by Matrix-Assisted Laser Desorption/Ionization Time-of Flight (MALDI-TOF) Mass Spectrometry.

### Structural characterization of recombinant proteins

The wild-type and mutant αB-crystallins were characterized by spectroscopic methods. All assays were performed in 50 mM phosphate buffer containing 150 mM NaCl and 0.02% sodium azide at pH 7.2.

### Tryptophan fluorescence

The intrinsic fluorescence spectra of wild-type and mutant αB-crystallin proteins were analyzed using a Jasco spectrofluorimeter FP-750 (JASCO Corporation, Tokyo, Japan). Protein samples at 0.2 mg/ml were excited at 295 nm, and the emission spectra were recorded between 300 and 400 nm.

### 1,1'-bi(4-anilino) naphthalene-5,5′-disulfonic acid fluorescence

The surface hydrophobicity of wild-type and mutant αB-crystallin proteins was compared using the probe, 1,1'-bi(4-anilino)naphthalene-5,5′-disulfonic acid (bis-ANS; Molecular Probes Inc., Eugene, OR). Bis-ANS (10 μl of 1 mM) was added to a 0.2 mg/ml sample and excited at 385 nm, and the emission spectra were recorded from 400 nm to 600 nm.

### Circular dichroism studies

Changes in protein secondary structure were evaluated by far- and near-ultraviolet (UV) circular dichroism (CD) spectra in a JASCO J-815 CD spectrometer (JASCO Inc., Easton, MD). A protein concentration of 0.2 mg/ml was used for far-UV CD measurements, and a concentration of 3 mg/ml was used for near-UV CD measurements. At least six scans in 5 mm path lengths were recorded for each sample and then averaged. Secondary structural elements were determined according to a computer software program derived from Sreerama and Woody [33].

### Molecular size determination by multi-angle light scattering

The quaternary structure of the recombinant αB-crystallin and its mutants was determined by multi-angle light scattering measurements. Protein samples (100 µg in phosphate buffer) were passed through a TSK G5000PW_XL_ gel (Tosoh Bioscience, Montgomeryville, PA) size-exclusion column connected to a HPLC system with refractive index detector (Shimadzu Scientific Instruments, Inc.,Columbia, MD) and a multiangle light scattering and quasi-elastic light scattering detectors (Wyatt Technology Corp., Santa Barbara, CA). The molar mass, hydrodynamic radius and polydispersity of the samples were estimated using ASTRA (5.1.5) software, as described previously [34].

### Electron microscopic study

The oligomeric structure of the mutant αB-crystallin proteins were examined under the JEOL 1200EX electron microscope (JEOL, Ltd. Tokyo, Japan). A drop of protein (1 mg/ml) was applied to carbon-coated grids and negatively stained with 2% uranyl acetate, and the specimens were examined under different magnifications [8].

### Functional characterization of recombinant proteins

For aggregation studies, 250 μg of alcohol dehydrogenase (Biozyme Laboratories, San Diego, CA) was incubated at 37 °C in 1 ml of 50 mM phosphate buffer (pH 7.4), which contained 150 mM NaCl. Aggregation was initiated by the addition of 100 mM EDTA in the absence or presence of mutant or wild-type proteins. The extent of aggregation was measured by monitoring the light scattering at 360 nm in a Shimadzu UV-VIS spectrophotometer (Shimadzu Corporation, Kyoto, Japan) [3,32].

### Subunit interaction study by FRET assay

The interaction between αBI5C and αBT162C was measured using the FRET technique [15]. The cysteines in αBI5C and αBT162C were labeled with thiol reactive dyes. Alexa Fluor 488 (C_5_-maleimide) and Alexa Fluor 555 (C_5_-maleimide) were used as donor and acceptor fluorophores, respectively. Additionally, in some experiments, the proteins were labeled with amine reactive dyes, Alexa Fluor 350 (carboxylic acid TFP ester) as the donor fluorophore and Alexa Fluor 488 (carboxylic acid TFP ester) as the acceptor fluorophore as described previously [15]. For FRET assays, 50 µg of each labeled protein was incubated at 37 °C. Subunit exchange interactions were monitored by measuring the ratio of acceptor fluorescence intensity at baseline and at timed intervals (F_t_/F_0_), as described in a previous study [35]. The subunit exchange rate was calculated according to Bova et al. [28].

### Mass spectrometry analysis

The interaction between αBI5C and αBT162C was also studied by nanospray Quadrupole  Time of  Flight  Mass  Spectrometry. Reduced I5C and T162C proteins (25 μg each) were mixed together and incubated at room temperature for 3 h. Reducing agents were removed by the dialysis method, and the proteins were allowed to form dimers at room temperature. The sample was desalted using C-18 spin columns (Pierce Biotechnology, Rockford, IL), eluted in 70% acetonitrile, and analyzed by nanospray mass spectrometry to identify hetero-dimer mass.

## Results

### Expression, purification and characterization of the αB-crystallin mutants

**Figure 1:**
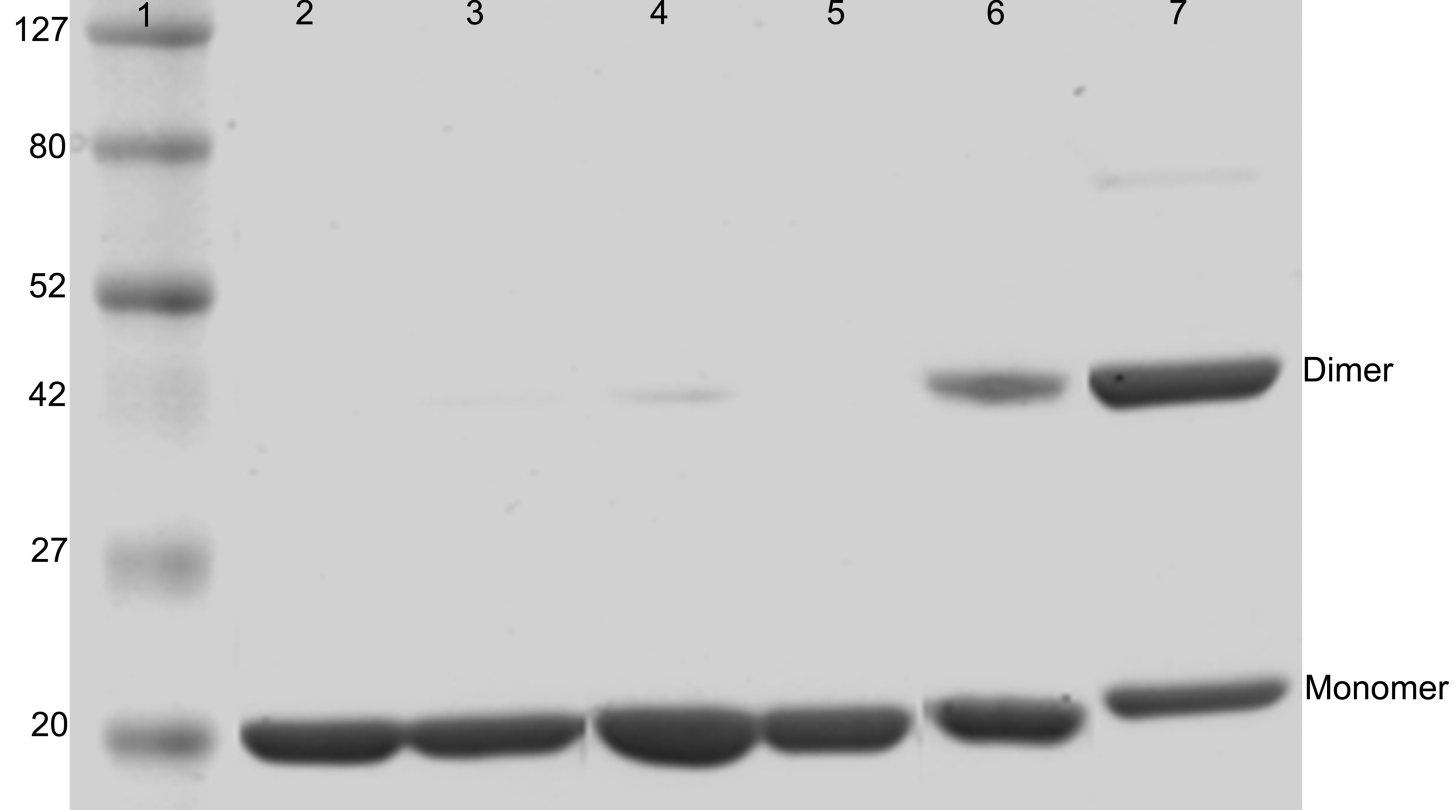
SDS-PAGE of αBI5C and αBT162C mutant and wild-type αB-crystallins under different conditions. Lane 1 protein markers in kDa; lanes 2 and 5 show the wild-type αB-crystallin; lanes 3 and 6 show αBI5C; lanes 4 and 7 show αBT162C; lanes 2, 3, and 4 show proteins under reducing conditions; and lanes 5, 6, and 7 show proteins under non-reducing conditions. Both mutants form dimers under non-reducing conditions and the higher intensity of αBT162C dimer band than that of αBI5C dimer suggests that the COOH-terminal extensions in the subunits interact more compared to the interactions between NH_2_-terminal regions.

Mutations were introduced in αB-crystallin either at the COOH-terminal extension or the NH_2_-terminal region to investigate the subunit interaction during homo-oligomerization. SDS–PAGE analysis of αBI5C and αBT162C proteins under reduced conditions showed that both proteins were more than 99% pure as was the case for the wild-type αB-crystallin. When αBI5C and αBT162C were stored at 4 °C in the absence of the reducing agent, TCEP·HCl Tris(2-Carboxyethyl) phosphine Hydrochloride (TCEP), and subjected to SDS–PAGE under non-reducing conditions and image analysis of the Coomassie blue stained gel was performed (Kodak ID image analysis software, Eastman Kodak Company, Rochester, NY), about 60% of αBT162C was present as dimers whereas only 25% of αBI5C was converted to dimers (Figure 1). SDS–PAGE of stored proteins under reducing conditions gave a profile similar to that of freshly purified proteins (not shown).

**Figure 2:**
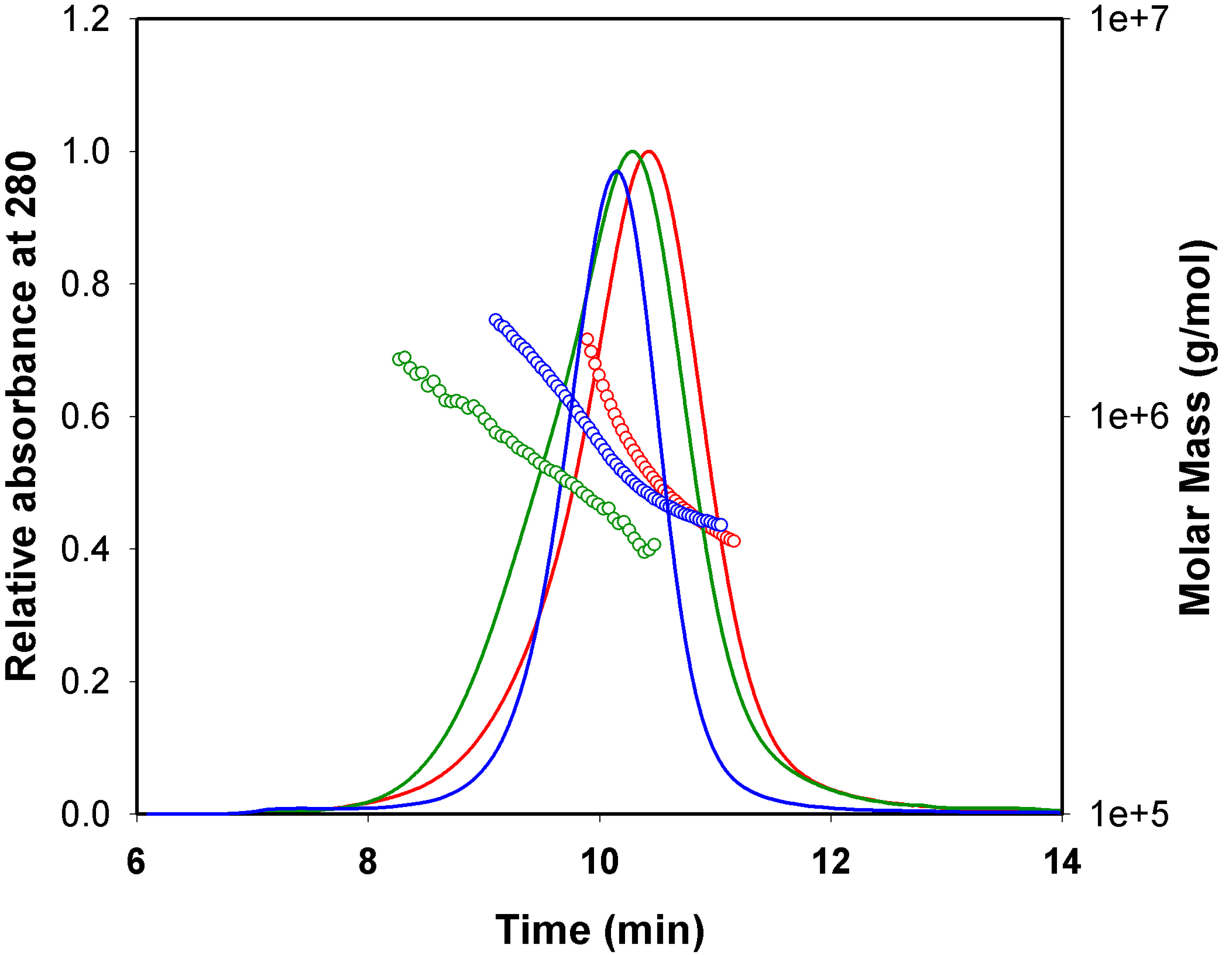
Molar mass distribution and elution profile of mutant and wild-type αB-crystallin proteins under reducing conditions. Protein (0.1 mg each) in buffer was injected into a TSK5000 gel filtration column connected to a multi-angle light scattering instrument, and the data was analyzed as described under Methods. Blue, wild-type αB-crystallin; Green, αBI5C; Red, αBT162C. Molecular mass of αBI5C and αBT162C mutants was not significantly altered compared to that of wild-type αB-crystallin under reducing conditions.

**Figure 3:**
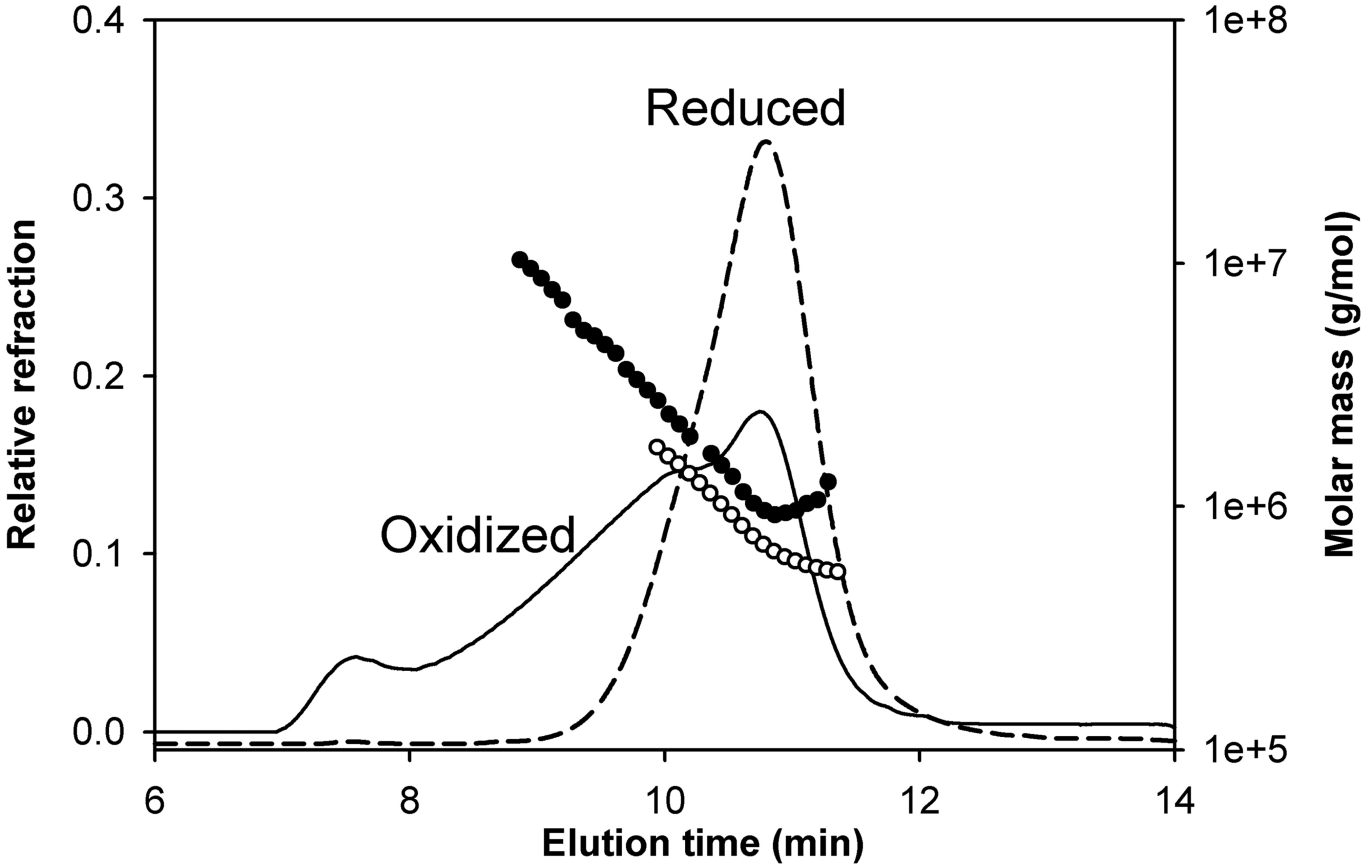
Molecular mass distribution and elution profile of oxidized and reduced αBT162C protein. Protein (0.1 mg each) in buffer was injected into a TSK5000 gel filtration column connected to a multi-angle light scattering instrument, and the data was analyzed as described under Methods. Solid line and filled circle, oxidized αBT162C; broken line and unfilled circle, reduced αBT162C. A broad elution peak for the oxidized αBT162C protein suggests that the cysteine mutant forms heterogeneous aggregates with wide-range of molecular species upon oxidation.

**Figure 4:**
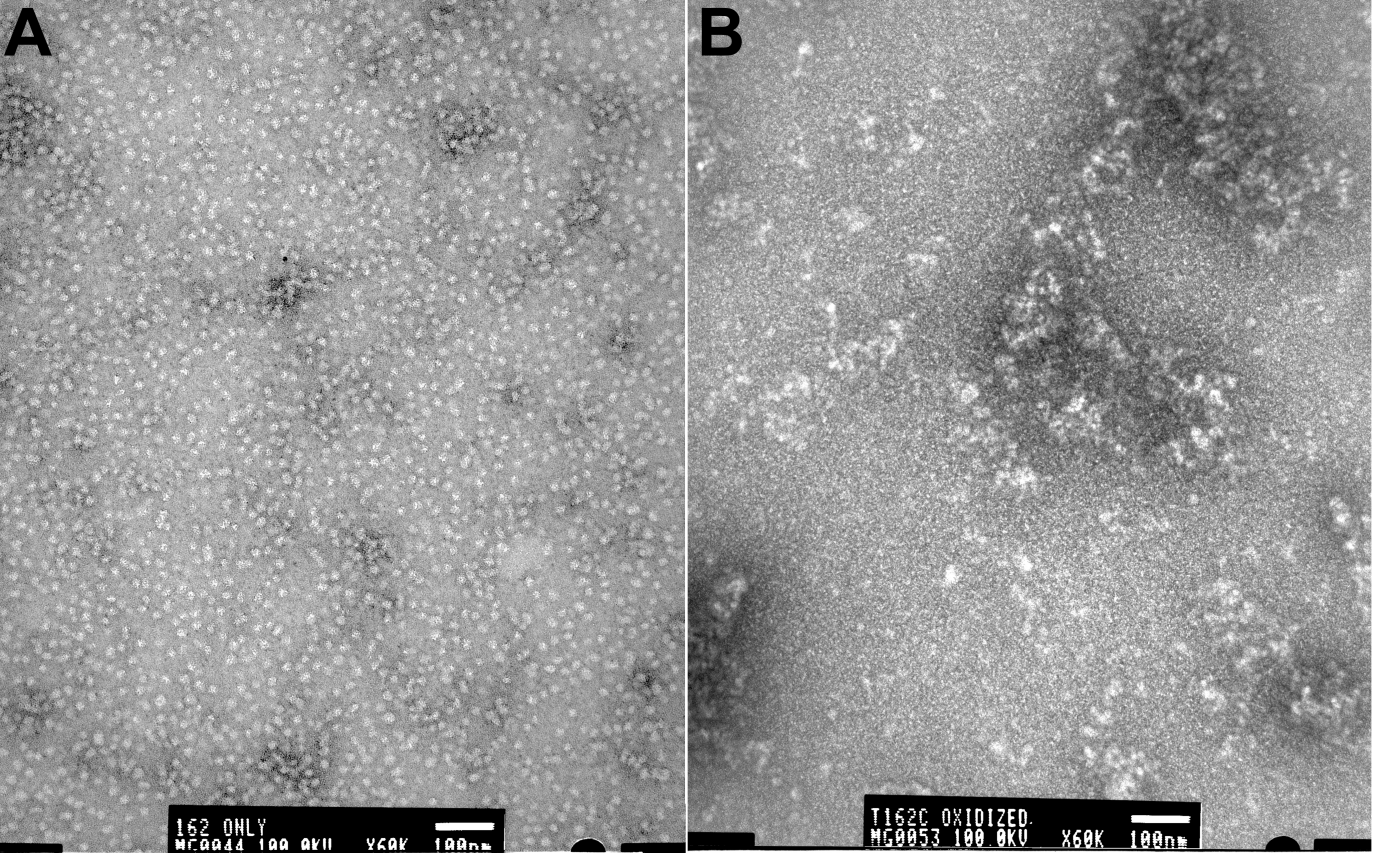
Electron microscopic image of αBT162C in reduced and oxidized form. A drop of 1 mg/ml protein was negatively stained with 2% uranyl acetate and observed under the JOEL 1200EX electron microscope. **A**: Reduced form shows homogeneous population of oligomers of 10-15 nm. **B**: Oxidized αBT162C forms highly heterogeneous and larger aggregates. The EM analysis of reduced and oxidized αBT162C confirms the aggregation of αB-crystallin following oxidation and crosslinking.

Gel chromatography by TSK 5000 column in the presence of the buffer that contained reducing agent TCEP and analysis of the eluted proteins by multi-angle light scattering methods revealed that both wild-type and mutant proteins exhibit similar elution profiles with only a minor difference. The wild-type and mutant forms of αB-crystallin showed an average oligomeric mass of 700±130 kDa at the peak apex (Figure 2), indicating that the mutation did not significantly affect the homo-oligomerization of the proteins. This suggested that on average the oligomers were composed of 35 subunits. In contrast, in the absence of the reducing agent in the buffer, both αBI5C and αBT162C mutants formed larger oligomers, presumably due to the cysteine–cysteine cross-linking of subunits or oligomers. The TSK 5000 column profile shown in Figure 3 for oxidized and reduced αBT162C crystallin indicates the heterogeneity in the oligomers. Under similar elution conditions, αBI5C stored without reducing agents was found to elute as a broad peak (not shown), which suggests that the oligomers further aggregate. Under electron microscopy, mutant αBI5C and αBT162C proteins stored in the absence of TCEP appeared as aggregates with irregular shapes whereas the oligomeric assembly of the reduced forms of αBI5C and αBT162C was more homogenous (Figure 4) with a diameter of about 10–15 nm, which is comparable to that of wild-type αB-crystallin [9].

**Figure 5:**
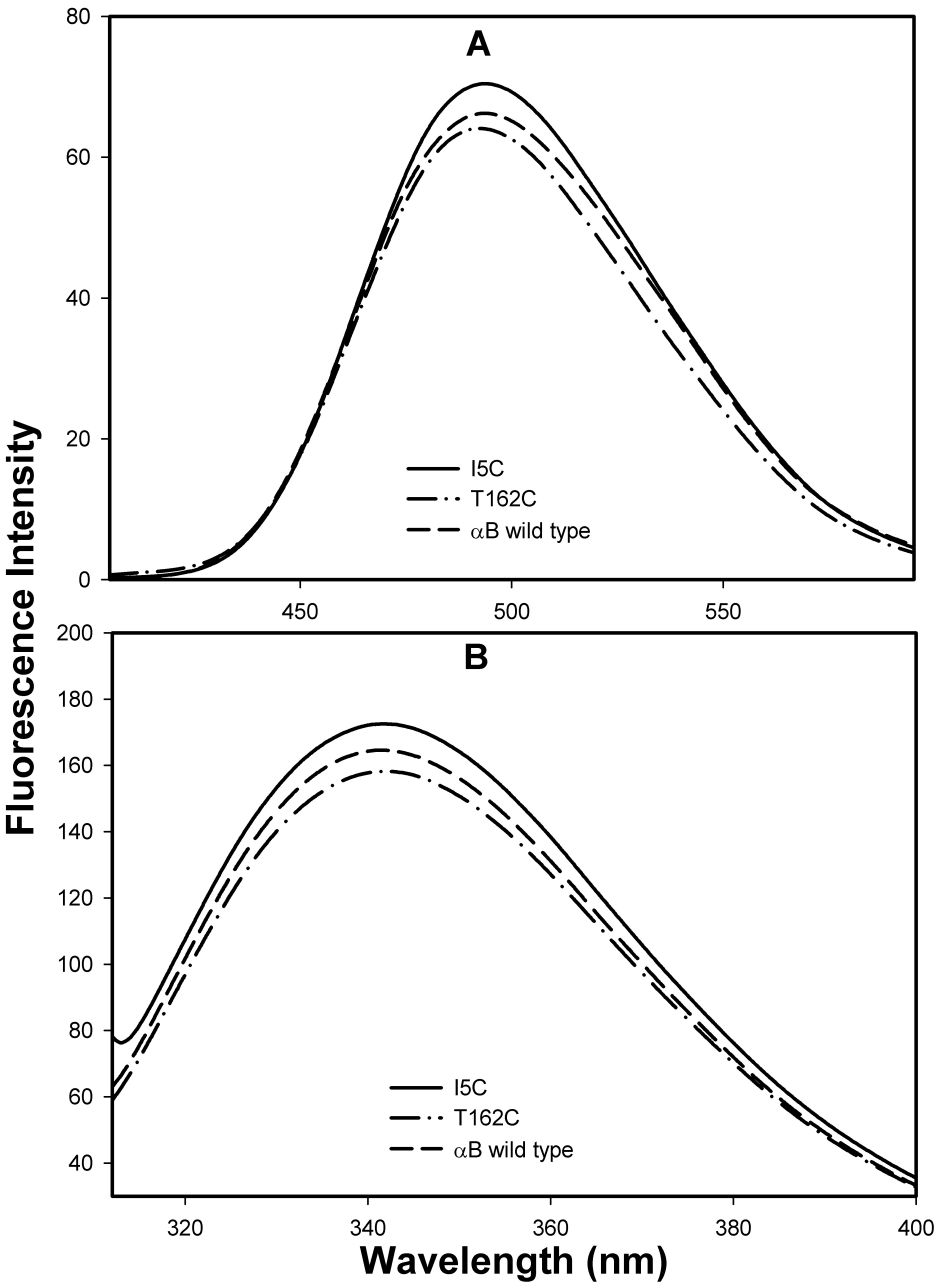
Bis-ANS binding and intrinsic tryptophan fluorescence profile of mutant αBI5C and αBT162C proteins. **A**: Bis-ANS 10 μl (1 mM) was added to protein samples of 0.2 mg/ml in 50 mM phosphate buffer (pH.7.2) were excited at 385 nm, and emission was scanned between 400 nm and 600 nm. The mutant αBI5C and αBT162C proteins show similar bis-ANS intensity like wild-type αB-crystallin. **B**: The intrinsic tryptophan fluorescence spectra of wild-type αB-crystallin, αBI5C and αBT162C. Protein samples (0.2 mg/ml) in 50 mM phosphate buffer (pH 7.2 ) were used. The samples were excited at 295 nm and the emission was scanned between 310 nm and 400 nm. Tryptophan spectrum of mutant αBI5C and αBT162C proteins were comparable to the wild-type αB-crystallin spectrum. The data suggests that the mutation did not alter the native conformations of mutant proteins under reducing conditions.

**Figure 6:**
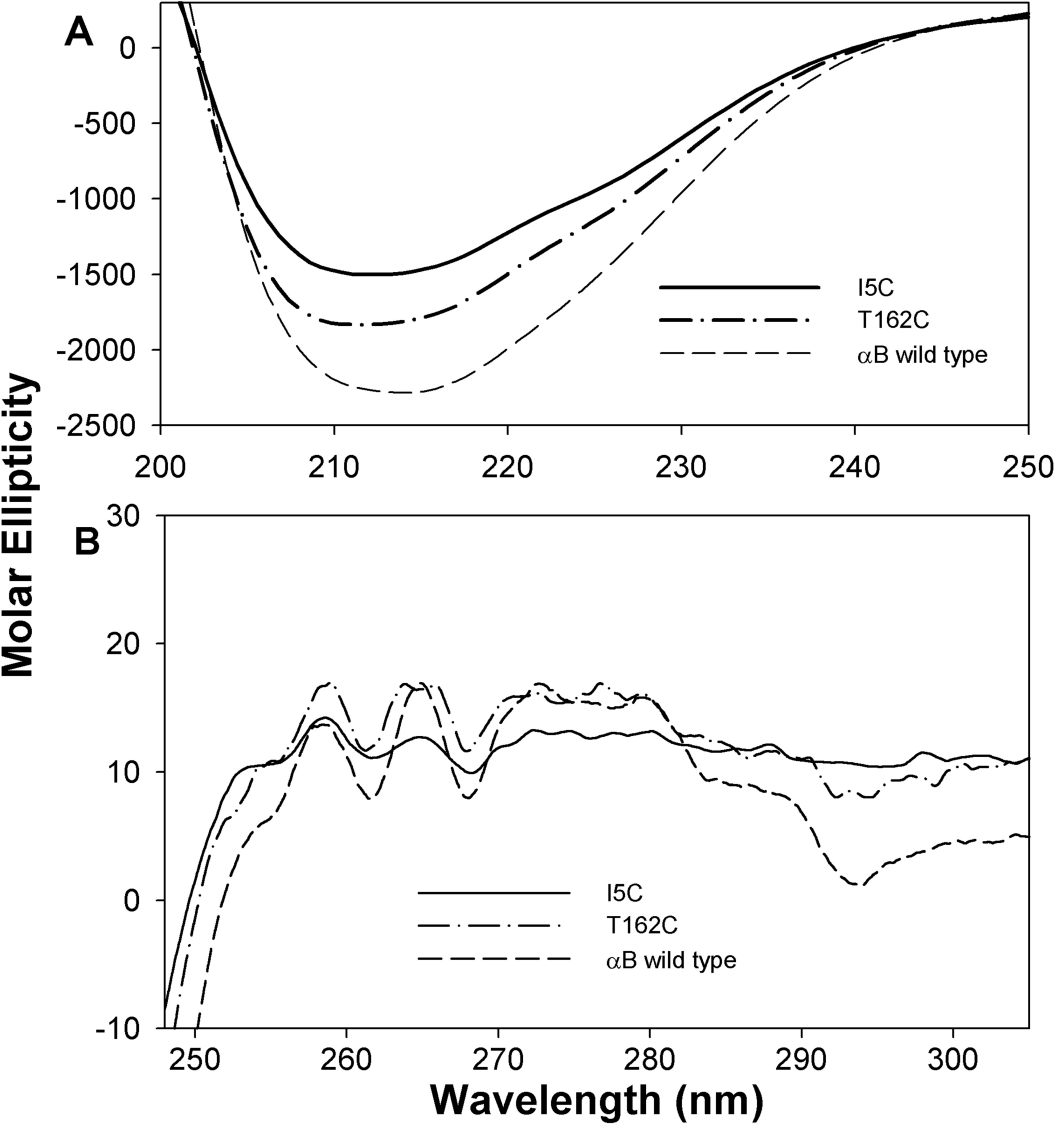
Circular dichroism spectra of wild-type and mutant I5C and T162C crystallins. **A**: Far-UV CD spectra were recorded using 0.2 mg/ml protein in a 0.5 cm cell path length at 25 °C. **B**: Near-UV CD spectra of wild type and mutant were recorded using a protein sample of 3 mg/ml in 0.5 cm cell path length at 25 °C. Both far- and near-UV CD spectra of αBI5C and αBT162C show negligible differences in secondary and tertiary structures. The spectra under reducing conditions are similar to that of wild-type αB-crystallin. These results further confirm the minimal impact of αBI5C and αBT162C mutations on the structure of αB-crystallin.

**Figure 7:**
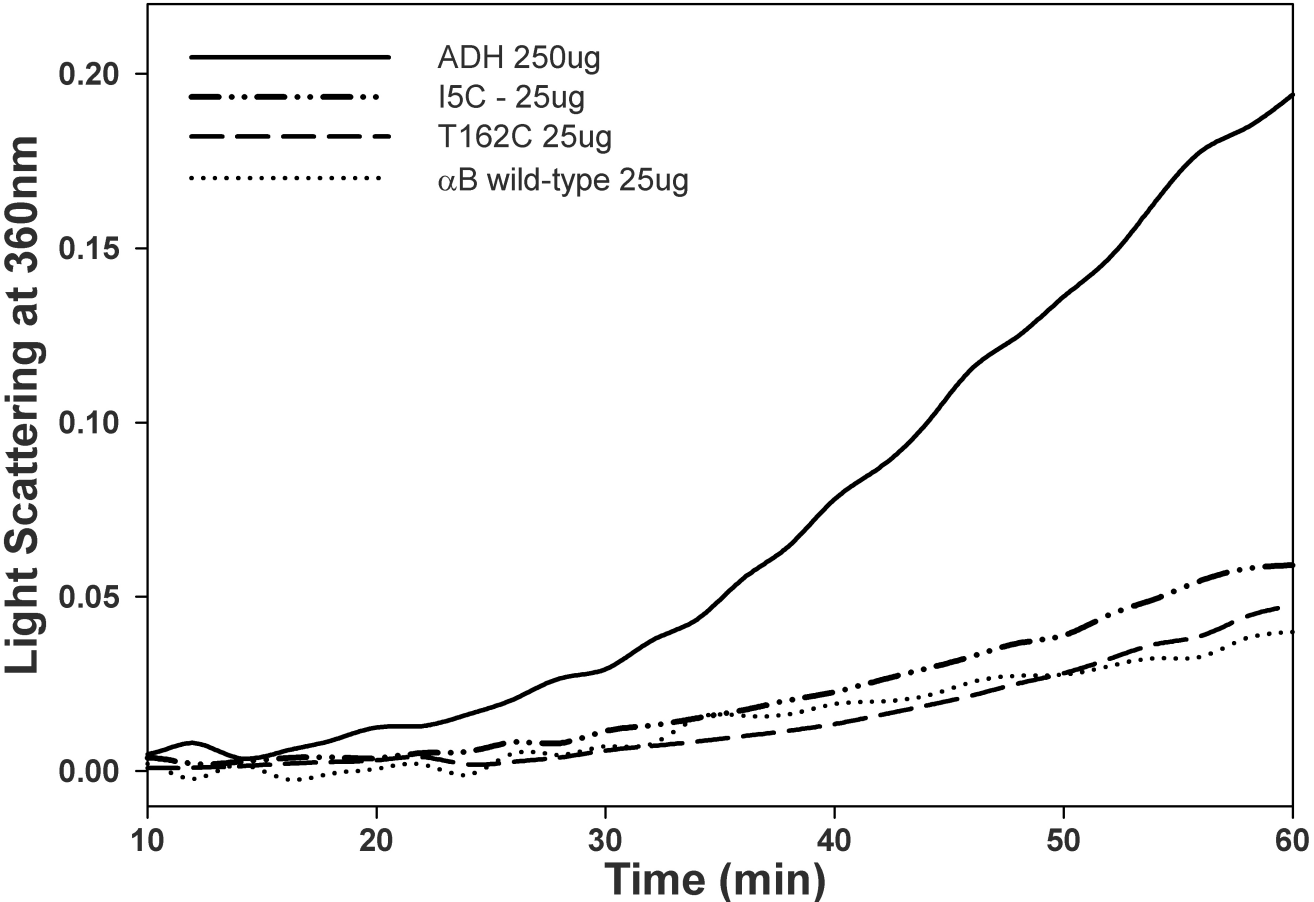
Chaperone activity of αBI5C and αBT162C proteins and wild-type αB-crystallin. EDTA-induced aggregation of ADH in the absence or presence of wild-type or mutant proteins was measured at 37 °C. In these experiments, 250 µg of ADH was used with or without crystallins. The chaperone activities of the mutants and wild-type proteins were similar when ADH was used as client protein. This study indicates that the mutation did not alter the structure-function of the protein maintained under reducing condition.

Intrinsic tryptophan fluorescence and bis-ANS fluorescence of the αBI5C and αBT162C proteins under reducing conditions were similar to those of wild-type αB-crystallin (Figure 5). Secondary structure conformations of reduced αBI5C and αBT162C showed increased ellipticity compared to that of the wild-type αB-crystallin as determined by circular dichroism (Figure 6A). The near-UV CD spectra for the mutants also showed increased chirality in the aromatic region, suggesting greater packing (Figure 6B). We also investigated whether the αBI5C and αBT162C mutations altered the chaperone-like function of αB-crystallin. EDTA-induced aggregation of alcohol dehydrogenase (ADH) at 37 ^°^C was prevented by both mutant αBI5C and αBT162C proteins, and the activity was comparable to that of the wild-type αB-crystallin (Figure 7). However, cysteine mutants stored without TCEP showed decreased chaperone-like activity as in the case of αBS59C and αBS19C reported earlier [36]. The two mutants stored in a buffer containing a reducing agent showed about the same amount of chaperone activity as the wild-type protein when citrate synthase was used as the substrate (data not shown).

### Subunit interactions on fluorescence energy transfer studies

**Figure 8:**
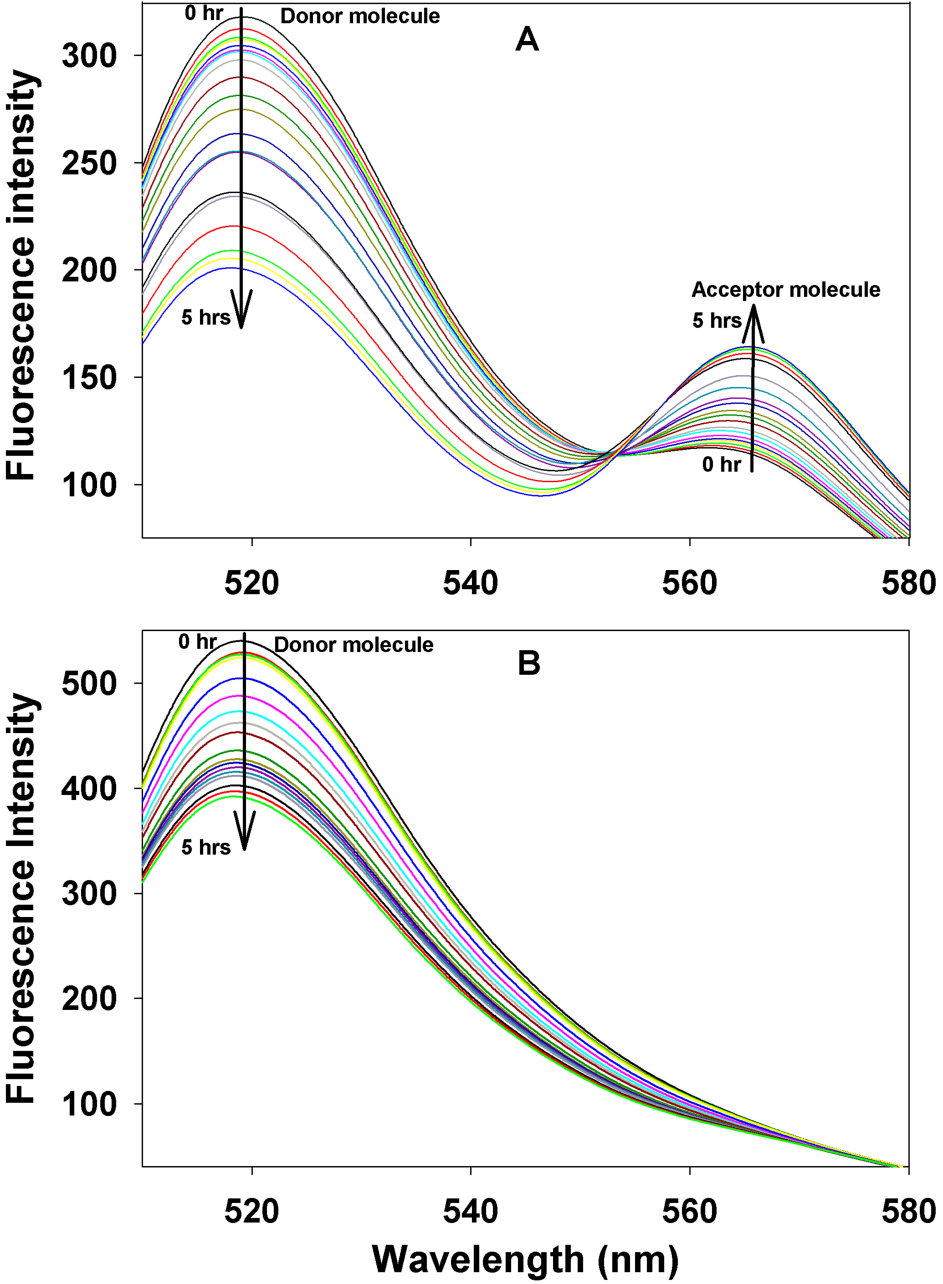
Subunit interaction determined by FRET assay. **A**: Time-dependent changes in the emission spectra of donor and acceptor Alexa Fluor dyes (thiol) that were labeled in αBT162C due to FRET are shown. The emission spectra of αBT162C, excited at 485 nm, were recorded at 5–20 min intervals after mixing an equal amount of donor and acceptor protein at 37 °C. The arrow marks show that donor fluorescence intensity decreases at 519 nm and acceptor emission increases at 565 nm. Emission spectra were recorded for 5 h with the excitation wavelength of 495 nm. **B:** There was a time-dependent decrease in the emission spectra of thiol-reactive, Alexa Fluor-labeled αBI5C in the presence of Alexa Fluor-labeled αBT162C. Note the absence of Alexa Fluor spectra with 565 nm maximum indicating that there is no energy transfer to the acceptor Alexa Fluor 555 from the donor Alexa Fluor 488. The results suggest that there was no interaction between cysteines in NH_2_- and COOH-terminal regions of the αB-crystallin subunits.

**Figure 9:**
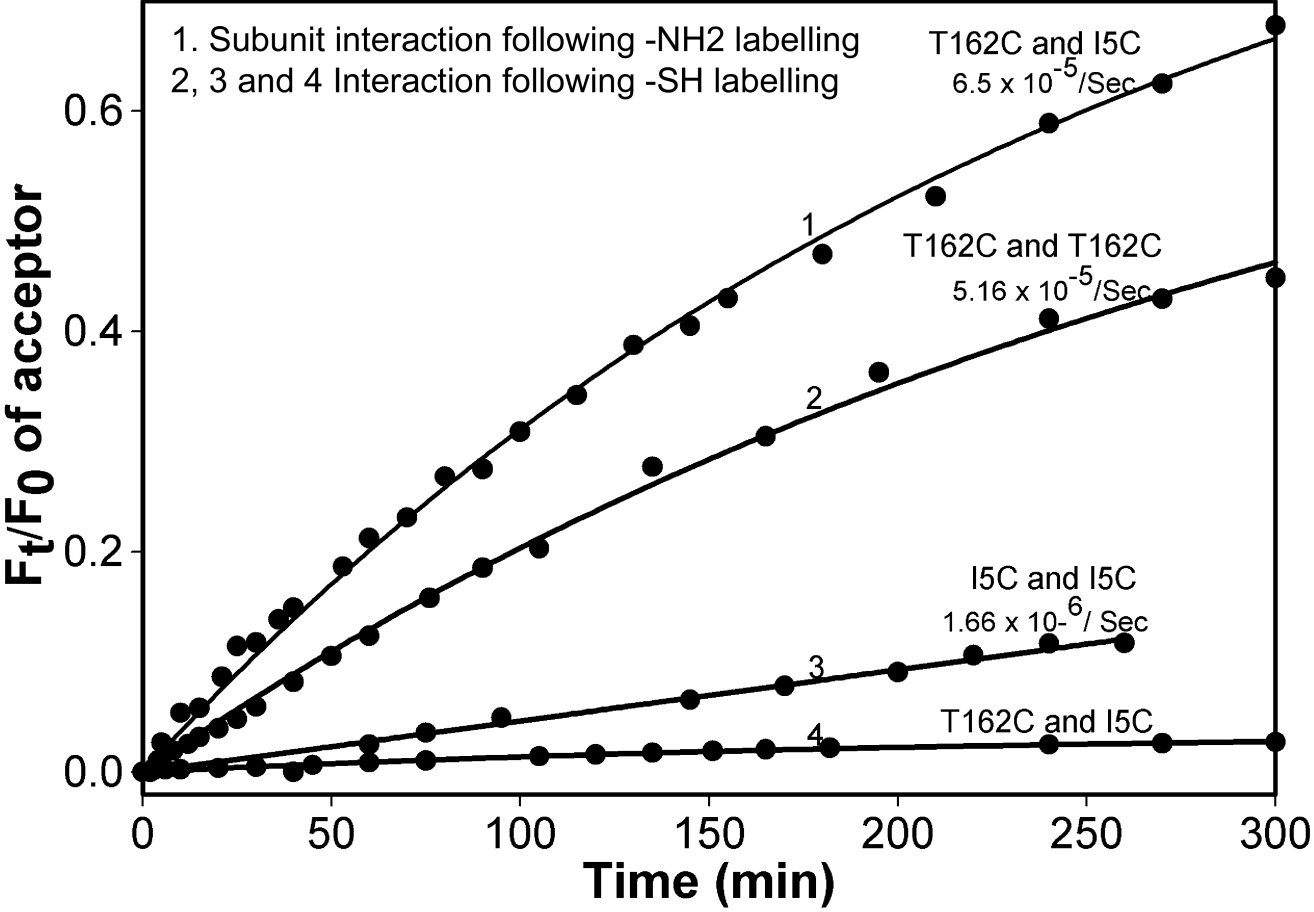
Subunit exchange studies with αB-crysteine mutants. FRET assay was performed with 50 μg of each donor and acceptor Alexa Fluor labeled protein at 37 ^°^C. The rate of subunit exchange was calculated by measuring the increase of acceptor fluorescence intensity. Ft/F0 represents the ratio of acceptor fluorescence intensity at given time intervals to the fluorescence intensity at the baseline. Curve 1 is the NH_2_-labeled protein; Curves 2, 3, and 4 are thiol-labeled proteins. The rate of subunit exchange between NH_2 _-labeled αBT162C–αBI5C was 65.5x10^-5^sec^-1^. The interaction rate between thiol-labeled αBT162C subunits was 5.16x10^-5^sec^-1^. The rate of interaction between thiol-labeled αBI5C subunits was 1.66x10^-6^sec^-1^. There was no measurable interaction between αBT162C and αBI5C when one of the mutants was labeled with a donor Alexa Fluor and the second mutant was labeled with an acceptor Alexa Flour at thiol residues. The results demonstrate the absence of interaction between NH_2_- and COOH-terminal regions of the two subunits and the presence of high degree of interactions between two NH_2_-terminal regions or two COOH-terminal regions of  αB-crystallin in an oligomer.

Fluorescence labeling has been used previously to investigate αA- and αB-crystallin interactions [37]. Previous studies have shown that the labeling of αB-crystallin with Alexa Fluor does not affect the properties of the protein [37]. Therefore, we used a similar approach to investigate αB-wild-type and mutant interactions. In these studies, the reaction mixtures of Alexa Fluor 488–αBT162C and Alexa Fluor 555–αBT162C (both labeled at Cys) showed a time-dependant decrease in the emission intensity of donor fluorophores at 519 nm and a relative increase in the emission intensity of acceptor fluorophores at 565 nm, indicating close proximity of the two labeled residues (Figure 8A). The interaction rate for αBT162C–αBT162C was 5.16×10^−5^ sec^−1^. A similar assay of αBI5C labeled with Alexa Fluor 488 as donor and Alexa Fluor 555 as acceptor revealed a measurable interaction between the subunits, and the rate of subunit interaction was 1.66×10^−6^ sec^−1^. The difference in the calculated rate in subunit interaction between I5C and T162C mutant proteins is likely a reflection of the proximity of Cys groups involved in interaction. The T162C mutant, which showed greater propensity to form dimers upon oxidation, also gave a better value of interaction during FRET assay. Surprisingly, in an experiment in which we mixed oligomers of αBI5C and αBT162C labeled with Cys-reacting fluorophores, Alexa Fluor 488 and Alexa Fluor 555, respectively, negligible fluorescence resonance energy transfers occurred between the two labeled proteins (Figure 8B). It appeared as though there was no subunit interaction between the two sets of oligomers. However, when the interactions were reexamined using amine-labeled αBI5C (donor) and αBT162C (acceptor), the transfer of energy was robust from the donor to the acceptor. The calculated subunit interaction rate for amine-labeled experiments was 6.5×10^−5^ sec^−1^ (Figure 9), higher than observed for cysteine-labeled αBI5C or αBT162C. The absence of interaction between I5C and T162C of αB-crystallin was further confirmed by mass spectrometric analysis of a mixture of the two proteins. Nanospray QqTOF MS analysis of a mixture of αBI5C and αBT162C showed a molecular mass of 20149.6 and 40299.45, corresponding to the monomers and dimers of αBI5C, respectively, and molecular mass of 20160.8 (monomer) and 40323.08 (dimer) of αBT162C. However, there was no evidence of a hetero-dimer having a mass of 40308.43, which corresponds to the mass of αBI5C-αBT162C dimer, suggesting that the two cysteine mutants do not form dimers involving cysteines from hetero subunits.

## Discussion

Several models of the quaternary structure of α-crystallin have been proposed including a dynamic protein micelle model [38], a cubic and rhombic dodecahedron model [39], a pitted-flexiball model [40], a chaperone-like annulus or toroid model [7], an octomer disc-like shaped model [41], and an open-loose model [42]. No general agreement has been reached for a consensus model that incorporates the dynamic and polydispersed nature of this heteromeric protein. However, due to their sequence similarity to αA- and αB-crystallin, the three-dimensional structures of a sHSP from Mj HSP16.5 [12] and wheat HSP 16.9 [13] make it possible to model the quaternary structure of α-crystallin. On the basis of crystal structure data on Mj HSP16.5 [12] and wheat HSP16.9 [13] and cryo-EM data on αB-crystallin, it has been proposed that αB-crystallin subunits assemble to form different molecular mass oligomers with a large central cavity [43,44]. We indeed observed under electron microscopy a less dense central region in both αBI5C and αBT162C crystallin oligomers, suggesting the presence of a lightly packed cavity. While the significance of the cavity in the α-crystallin oligomer is not known, the arrangement of subunits with a central cavity offers the flexibility to accommodate a varying number of subunits in a dynamic oligomer.

The flexible COOH-terminal hydrophilic tail is likely to be important for keeping the protein in soluble form [7]. The more hydrophobic region of the NH_2_-terminal domain would favor multimerization of αB-crystallin [11,14]. Electron paramagnetic resonance studies combined with site-directed spin labeling studies revealed that αB-crystallin is composed of flexible building units with an extended surface area where β4−β8 strands of the protein are involved in subunit–subunit interaction during complex assembly [10,44]. However, those studies did not include the extent of the interaction between two NH_2_-terminal extensions  or  two COOH-terminal regions extension of αB-crystallin during oligomerization. In a study where Hsp16.5 was converted to a polydisperse oligomer by insertion mutagenesis [44], the COOH-terminal was found to tether between the dimers. Further, the NH_2_-terminal of the engineered protein was more exposed and available for interaction with denaturing proteins [44]. We used the Cys–Cys cross-linking approach to understand how the NH_2_- and COOH-terminal regions in αB-crystallin interact during oligomerization. Since human αB-crystallin is a Cys-free protein, we introduced a single Cys at the COOH-terminal and NH_2_-terminal regions to obtain αBT162 and αBI5C mutants. The introduction of Cys at these two positions did not affect chaperone activity (Figure 7), overall hydrophobicity (Figure 5A), or intrinsic tryptophan fluorescence (Figure 5B). The Cys mutants showed only minimal structural changes (Figure 6) and oligomer size (Figure 2). Together, these findings suggest that the structure-function of the mutants did not change significantly from that of the wild-type protein.

The labeling of αBI5C and  αBT162C  mutants  with fluorescent probes Alexa Fluor 488 and Alexa Fluor 555,  respectively, did not disrupt the oligomer size and function of the proteins. The results of the FRET assay suggest that the αBT162C residue of a subunit comes into close proximity of αBT162C of another subunit (Figure 8A). The distance between donor and acceptor molecules should remain less than 100 Å for a successful energy transfer [45]. Similarly, FRET studies showed that 5C of one subunit comes in close proximity of 5C from another αB subunit during oligomerization. In contrast, the FRET assay demonstrates no energy transfer between αB5C (donor) and αB162C (acceptor), suggesting that the I5 residues at the NH_2_-terminal region of a subunit and the T162 residues at the COOH-terminal region of another subunit do not interact during oligomerization. However, the αBI5C protein labeled at Lys residues and the αBT162C crystallin readily exchange subunits during oligomerization. This was evident from the FRET between the NH_2_-labeled I5C (donor) and labeled αBT162C (acceptor), and the rate of subunit exchange was maximal, reflecting unhindered interaction and fluorescence energy transfer. The data further confirm that the mutation did not affect the subunit exchange. There are 10 lysine residues in αB-crystallin, and the majority of these are in the α-crystallin domain [11]. Among these residues, αBK82, αBK90, αBK92, αBK103, αBK119, and αBK150 are at the subunit interaction regions in αB-crystallin homo-oligomer [27]. It is likely that the labeling at these sites might have led to maximal energy transfer between amine-labeled αBI5C and αBT162C proteins. Chemical cross-linking has been used previously to determine the interactions between αA-crystallin and αB-crystallin in a hetero-oligomer [25,26]. In one study, αA-crystallin Lys166 cross-linked to αB-crystallin Lys175 when 3.3′-dithiobis[sulfosuccinimidyl propionate] (DTSSP) was used as the cross-linker [26]. In another study, however, the same residues were not cross-linked by DTSSP, but it was suggested that Lys150 might be cross-linking with Lys166 either intramolecularly or intermolecularly [25]. The discrepancy between the results of these two studies may be due to differences in experimental conditions and sample preparations. Additionally, since α-crystallin is composed of αA- and αB-crystallin in approximately 3:1 ratio [11], it is likely that the mosaic nature of the heteroaggregate does not permit cross-linking between αB-crystallin subunits. In support of this view, we observed that when αA-crystallin was mixed with αBT162C in equal ratio, the cross-linking of αB-crystallin mutants decreased significantly (not shown). Previous cross-linking studies [25,26] have not shown NH_2_-terminus interactions between αB-crystallin subunits, but Lys11 of αA-crystallin was suggested to be involved in interactions with Lys121 of αB-crystallin [25]. A two-hybrid study was also performed to determine the involvement of COOH- and NH_2_-terminal regions of αB-crystallin [20]. Contrary to the observation made in our study, the two-hybrid system assay showed that only the COOH-terminal domain was important for self aggregation of αB-crystallin. Since it is also known that αB-crystallin interacts with other proteins through its chaperone site, it is not clear to what extent the chaperone action was involved in the two-hybrid system interaction studies interpreted as subunit interactions. Additionally, it is not known whether the hybrid proteins used in the study with NH_2_- or COOH-terminal domains of αB-crystallin folded the same way as in the native protein where the subunit interaction sites are fully accessible.

For the first time, our study of Cys-substituted mutants, which did not involve the use of cross-linking agents, demonstrated the involvement of residue(s) close to the NH_2_-terminus of αB-crystallin with corresponding residue(s) from another subunit during oligomerization. The NH_2_-terminal region comprising of 5C residues in the αB-crystallin mutant that we identified as the interacting region does not have an equivalent sequence in HSPs 16.5 and 16.9, the two proteins with known crystal structures [12,13]. Therefore, the homology model study of αB-crystallin has only provided structural insight to the αB-crystallin 20–170 region [21]. The results of the present study suggest that in αB-crystallin, a residue in the NH_2_-terminal 1–20 region has a role in oligomerization of this sHSP. Previous mutagenesis studies involving the conserved Phe-rich region in the NH_2_-terminal domain suggested that residues may be important for both structure and function of αA-crystallin [16]. Limited trypsin digestion studies [22] also suggested that the NH_2_-terminal region is involved in oligomerization of αB- and αA-crystallins. However, those studies did not indicate the role of specific residues in the NH_2_-terminal domain.

The involvement of the COOH-terminal T162 region of αB-crystallin in oligomerization was earlier identified by pin array studies as one of the interactive sequences [19]. However, the same study did not show the I5 region as an interacting site during αB-crystallin oligomerization. The pin array study also did not identify the region in αB-crystallin responsible for interactions with the peptide(s) in array. It should be noted that the lack of three dimensional (3D) conformation of peptides in a pin array does not offer a recognizable binding site similar to that presented by a folded protein. The interactions between the COOH-terminal extensions appear to be more pronounced than those between NH_2_-terminal regions because Cys oxidation that induced dimer formation was about three times more prevalent for T162C mutants than for I5C mutants under identical storage conditions. The mass spectroscopic study also suggested that the T162C forms a dimer more readily than the I5C. However, the mass spectroscopic studies did not demonstrate the interaction between I5C and T162C mutants of αB-crystallin through Cys residues. Previous studies have shown that αB-crystallin subunit interaction involves the NH_2_-terminal domain, the α-crystallin domain, and the COOH-terminal extension [15-19]. However, none of the studies found interaction between the NH_2_-terminal domain and COOH-terminal extensions, but there is evidence for the interaction between residues 108 and 110 in the α-crystallin domain and residues 7–10 in the NH_2_-terminal domain of HSP16.9 [13]. As discussed above, FRET studies involving I5C and T162C did not show any interactions between the epitopes carrying these mutations. Therefore, one can conclude that even though there are multiple interactions between the αB-crystallin subunits, they do not involve interactions between NH_2_-terminal residues 1–19 and COOH-terminal extensions. The aggregation of αBI5C or αBT162C (Figure 4) following oxidation suggests that forced dimerization of αB-crystallin subunits leads to aggregation and precipitation. Therefore, it is likely that cross-linking of subunits following age-related modifications such as glycation may result in the formation of light scattering aggregates in vivo. Recently, it was shown that the COOH-terminal truncation of αB-crystallin causes protein insolubilization and myopathy [46], and it was shown that the truncated proteins aggregate in vivo. Our study shows that the COOH-terminal residues play a role in oligomerization, and disruption of this interaction may be a causative factor in the destabilization and uncontrolled aggregation of the αBQ151X mutant described earlier [46]. Further, it is likely that age-related truncation of αB-crystallin may result in oligomers that are not governed by the physiologically significant COOH-terminal interactions. Instead, the crystallin subunits may form large oligomers that scatter light.
